# Intestinal microbiota and chronic constipation

**DOI:** 10.1186/s40064-016-2821-1

**Published:** 2016-07-19

**Authors:** Ying Zhao, Yan-Bo Yu

**Affiliations:** Department of Geriatrics, Jinan Military General Hospital, Jinan, 250031 People’s Republic of China; Department of Gastroenterology, Qilu Hospital, Shandong University, Jinan, 250012 People’s Republic of China

**Keywords:** Intestinal microbiota, Chronic constipation, Gut motility, Probiotics

## Abstract

Chronic constipation is a prevalent, burdensome gastrointestinal disorder whose aetiology and pathophysiology remains poorly understood and is most likely multifactorial. Differences in the composition of the intestinal microbiota have been demonstrated when constipated patients and healthy controls have been compared. Growing evidence indicates that alterations of intestinal microbiota may contribute to constipation and constipation-related symptoms. The intestinal microbiota is a collection of microorganisms that live within the gastrointestinal tract, and perform many important health-promoting functions. The intestinal microbiota aids in the breakdown of food products into absorbable nutrients, stimulates the host immune system, prevents growth of pathogenic bacteria and produces a great variety of biologically important compounds. In this review, we will summarize the current evidence supporting roles of the intestinal microbiota in the pathogenesis and management of chronic constipation. The discussion will shed light on the novel mechanisms of intestinal microbiota and gut function interactions, which is invaluable in ultimately developing new therapeutic tools for the treatment of chronic constipation.

Chronic constipation is a symptom-based gastrointestinal disorder characterized by difficult or infrequent passage of stool, hardness of stool, and/or a feeling of incomplete evacuation. It can result in some discomfort such as abdominal distension, abdominal pain, headache, dizziness and poor appetite. Constipation has an estimated prevalence reaching up to 20 % in some populations, and this condition affects patients of all ages and gender and severely impacts on their quality of life (Bharucha 2007). The aetiology and pathophysiology of chronic constipation remains poorly understood and is most likely multifactorial. Furthermore, the clinical management of chronic constipation remains challenging. Growing evidence indicates that alterations of intestinal microbiota may contribute to constipation and constipation-related symptoms, which attracts considerable interest among gastrointestinal researchers in recent years.

The intestinal microbiota is a collection of microorganisms that live within the gastrointestinal tract, with concentrations of up to 10^11^–10^12^ cells/g luminal contents. It is recognized that the number of bacteria within the gut is nearly 10 times that of the cells in the human body, including up to 1000 different bacterial species (Blaser et al. 2013). The microorganisms have a symbiotic relationship with their host, and perform many important health-promoting functions. The intestinal microbiota aids in the breakdown of food products into absorbable nutrients, stimulates the host immune system, prevents growth of pathogenic bacteria and produces a great variety of biologically important compounds, such as short-chain fatty acids (SCFAs) and neuro-modulatory substances. Multiples studies have revealed disturbances in the composition and stability of the gut microbiota in constipated patients compared with healthy controls. In this review, we will summarize the current evidence supporting roles of the intestinal microbiota in the pathogenesis and management of chronic constipation.

## Possible alterations of intestinal microbiota in chronic constipation

Using standard cultural and/or molecular approaches, quantitative difference among intestinal microbiota species has been extensively studied. Alterations of intestinal microbiota in patients with chronic constipation can be characterized by a relative decrease in obligate bacteria (e.g. *Lactobacillus*, *Bifidobacterium*, and *Bacteroides* spp.) and a parallel increase of potentially pathogenic microorganisms (e.g. *Pseudomonas aeruginosa* and *Campylobacter jejuni*) (Gerritsen et al. 2011; Kirgizov et al. 2001). These alterations may influence intestinal motility and secretory functions by changing the amount of available physiologically active substances and the metabolic environment of the gut. Khalif et al. reported that the levels of *Bifidobacteria* and *Lactobacillus* were significantly decreased in adult patients with constipation (Khalif et al. 2005). Patients with constipation predominant-irritable bowel syndrome (IBS-C) exhibited a significant increase of *Bacteroides* sp. and *Enterobacteriaceae* (Simren et al. 2013). Further, the concentrations of *Bifidobacteria*, *Clostridium leptum* and *Faecalibacterium prausnitzii* were decreased in these patients with IBS-C (Nourrisson et al. 2014). Meanwhile, one pediatric study indicated that *Clostridia* and *Bifidobacteria* were significantly increased in feces of constipated children. Besides, the *clostridium* species isolated from constipated children were different from those from healthy controls (Zoppi et al. 1998). Using 16S rRNA gene pyrosequencing method, Zhu et al. observed significantly decreased abundance in *Prevotella* and increased representation in several genera of *Firmicutes* in constipated patients compared with controls (Zhu et al. 2014). Butyrate-producing genera, such as *Coprococcus*, *Roseburia* and *Faecalibacterium*, tended to be increased in constipated patients (Pryde et al. 2002; Sokol et al. 2008). These studies indicated that constipation could be a consequence of the intestinal dysbiosis.

It should be noteworthy that discordances have been reported in the detection of microbiota alterations among patients with constipation. This discrepancy may be the result of different methods used for bacterial quantifications, the use of single samples and other factors (e.g. diet and phenotypic characterization of patients) (Simren et al. 2013). Furthermore, most studies assessed the fecal microbiota, which are readily accessible, but do not fully replicate profiles of the mucosal microbiota of the patients with constipation. The mucosal microbiota might affect epithelial and mucosal function to a greater degree than the fecal microbiota (Durban et al. 2012; Parkes et al. 2012). One recent study reported that the overall composition of the colonic mucosal microbiota was associated with constipation and genera from *Bacteroidetes* were more abundant in the colonic mucosal microbiota of patients with constipation. Meanwhile, the profile of the fecal microbiota was associated with colonic transit and methane production, but not constipation (Parthasarathy et al. 2016). More studies detailing higher resolution of the mucosal and fecal microbiota composition might expand our understanding of the relationships between intestinal microbiota and constipation.

Though widely used, the above cultural and/or molecular approaches could not assess the functional imbalance of intestinal microbiota in patients with constipation. Thereby, the function-based approach, based on the detection of specific metabolic activity expressed by a group of bacterial species, has been put forward in recent studies. Chassard et al. showed that the IBS-C microbiota was characterized by a high number of lactate- and H_2_-utilising sulphate-reducing bacteria compared with healthy controls, which could in turn influence colonic motility and visceral sensitivity and generate IBS symptoms (Chassard et al. 2012). Due to changes in metabolism output, the functional dysbiosis might have important clinical implications and provide a new dimension in management of chronic constipation.

## Possible role of the intestinal microbiota in gut motility

Most of our knowledge on the effect of intestinal microbiota on gut motility derives from studies in germ-free animals. Germ-free rats exhibited longer migrating myoelectric complex (MMC) intervals compared with conventional rats (Caenepeel et al. 1989). Further, the cecum of germ-free rats was enlarged and gastric emptying and colonic transit were delayed (Abrams 1977). Studies colonizing of conventional intestinal microbiota to germ-free rats revealed that *Lactobacillus acidophilus* and *Bifidobacterium bifidum* could reduce the MMC period and accelerate small intestinal transit, while *Micrococcus luteus* and *Escherichia coli* showed an inhibitory effect (Husebye et al. 2001). Barbara et al. put forward three mechanisms responsible for the effects of microbiota on intestinal motility (Barbara et al. 2005): (1) the release of bacterial substances or end-products of bacterial fermentation; (2) intestinal neuroendocrine factors; (3) mediators released by the gut immune response.

The bacterial endotoxin lipopolysaccharide may influence intestinal motility by delaying gastric emptying and inducing sphincteric dysfunction (Fan et al. 2001). Deconjugated bile salts, a kind of bacterial metabolite, may promote colonic motor responses and induce bile salt related diarrhea (Floch 2002). Short-chain fatty acids (SCFAs), such as butyrate, acetate, and propionate are produced in the colon by anaerobic bacterial metabolism of carbohydrates. SCFAs have been shown to stimulate ileal propulsive contractions by evoking prolonged propagated contractions and discrete clustered contractions. The possible mechanisms of SCFAs in gut motility may involve the intestinal release of 5-hydroxytryptamine (5-HT). In addition, SCFAs could directly stimulate the ileal and colonic smooth muscle contractility. Lactate can be quickly metabolized by specific bacterial species into butyrate or propionate. High concentration of butyrate may inhibit mucin secretion by intestinal goblet cells (Baxter et al. 2014). Further study indicates that butyrate may reduce stool volume by stimulating water and electrolyte absorption in the colon (Canani et al. 2011). The number of these lactate-utilising bacteria is obviously decreased tenfold in the faecal microbiota of IBS-C patients (Rajilic-Stojanovic et al. 2011). Human colonic gases produced by microflora may also be associated with changes in gut motility. For example, breath methane excretion in patients with slow-transit constipation was greater than in healthy subjects or patients with normal-transit constipation, supporting the idea that methane can slow gut transit (Attaluri et al. 2010). However, recent study reported that methane production was associated with the composition of the fecal microbiota, but not with constipation or colonic transit (Parthasarathy et al. 2016). Meanwhile, H_2_ accumulation has been postulated to account for the symptom of bloating and pain (Pimentel et al. 2000). Colonic H_2_S has been demonstrated to modulate peripheral pain-related signals, which may ultimately influence gut sensory-motor functions (Kawabata et al. 2007). Cell-envelope-associated multi-protein system, termed as “starch utilization system (SUS)”, is present in *Bacteroides* species such as *Bacteroides thetaiotaomicron* (Lammerts van Bueren et al. 2015). Increased number of *Bacteroides* species may be associated with production of organic acids in excess via fermentation, which may contribute to the symptoms of abdominal distension. Small intestinal bacterial overgrowth (SIBO) is a condition involves abnormal growth of endogenous bacteria in the small intestine resembling those normally found in the colon. The clinical manifestations of SIBO are wide ranging and include abdominal discomfort, bloating, diarrhea, weight loss and nutritional deficiencies (Gabrielli et al. 2013). Due to the inherent difficulties in measuring small bowel motility, few studies have illustrated a direct relationship between impaired small intestinal motility and SIBO. Roland et al. (2015) recently reported that patients undergoing wireless motility capsule with SIBO have significant delays in small bowel transit time as compared with those without. Future prospective studies are needed to further characterize SIBO and contributing pathophysiological mechanisms in small bowel dysmotility.

It has been hypothesized that intestinal microbiota could modulate gut motility through the release of neuroendocrine factors. For instance, the presence of somatostatin was firstly clarified in *Bacillus subtilis* (Lenard 1992). Germfree rats had increased gastrin-, serotonin-, and motilin-immunoreactive cells in selective areas of the gastrointestinal mucosa. Neuropeptide Y, an inhibitory neuropeptide, was found decreased in blood concentration after introduction of conventional intestinal microbiota in these rats (Husebye et al. 2001). These findings indirectly demonstrated that the intestinal microbiota could regulate endocrine cells and ultimately influence the gut motility. Besides, the effect of microbiota-derived SCFAs on colonic serotonin and motilin-containing enteroendocrine cells has also been proposed in the regulation of colonic physiology.

In immunocompetent hosts, the commensal colonic microbiota are major determinants of mucosal and systemic immunity. For example, *Bacteroides fragilis* can mediate the development of Foxp3^+^ Treg cells through the activation of Toll-like receptor (TLR)2 (Nutsch and Hsieh 2012). *Clostridium* species induce Foxp3^+^ Treg cells through the induction of transforming growth factor-β (TGF-β) (Ivanov and Littman 2010). Mediators released by colonic immune cells are known to modulate various digestive functions. Increased expression of IL-1β, IL-6 and tumor necrosis factor α (TNF-α) have been demonstrated in colitis rat model. IL-1β could cause a pronounced suppression of the synthesis and release of both acetylcholine and noradrenaline (Collins 1996). Synergistic interactions between IL-1 and IL-6 have been confirmed both pharmacologically and electrophysiologically (Atanasova et al. 2011). Similar observations were drawn with respect to the role of TNF-α to suppress noradrenaline release (O’Sullivan et al. 2009). *Clostridial* species and polysaccharide A derived from *Bacteroidetes* species strains induce IL-10 production and promote T-regulatory cell development (Suszko and Obminska-Mrukowicz 2013). Segmented filamentous bacteria, *Candidatus svagella*, are required for differentiation of T helper 17 (TH17) cells and induce luminal secretion of IgA (Ivanov and Littman 2010). Disruptions in colonic immunity can result in abnormal responses to luminal contents and this mechanism is suspected to be a component in the development of colonic motility disorder.

## Possible microbiota-based therapy on chronic constipation

### Dietary fibre

Dietary fibre has been recommended on an empirical basis for the management of chronic constipation. As is known, dietary fibres can promote the excretion of intestinal mucin by stimulating the capacity of mucosal protein synthesis (Derrien et al. 2010). Dietary fibre is broken down in the proximal colon and provides an energy yielding substrate for microbial fermentation. The result of this is to stimulate the growth of intestinal microbiota and contribute significantly to the stool dry weight. Besides, fibres could promote the excretion of bacterial fermentation products, such as SCFAs, which has the pro-motility effects (Jennings et al. 2009). Dietary fibre is also an important source of gas in the colon. Hydrogen, methane and carbon dioxide, which are principal end products of bacterial fermentation, can increase stool bulk and promote colonic transit (Lopez Roman et al. 2008). There is evidence indicates that dietary fibres (wheat bran, pea fibres) can modulate intestinal microbiota, including the stimulation of beneficial bacterial species and the suppression of pathogenic bacterial species (Chen et al. 2013).

### Prebiotics

Prebiotics are non-digestible substances that provide a beneficial physiological effect on the host by selectively stimulating the growth or activity of a limited number of favourable indigenous gut bacteria. Prebiotics stimulate the preferential growth of a limited number of health-promoting commensal flora already residing in the colon, such as *Lactobacilli* and *Bifidobacteria*. Examples of prebiotics include fructo-oligosaccharides, galacto-oligosaccharides and inulin. Fibre and fibre supplements employed in the treatment of constipation also exert prebiotic effects (Bouhnik et al. 2004). Prebiotics are subjected to bacterial metabolism in the colon, where they are transformed to lactic and short-chain carboxylic acids. It has been demonstrated that galacto-oligosaccharides seemed to promote the intestinal peristalsis and relieve constipation (Li et al. 2013). The consumption of inulin-type fructans affects intestinal microbiota and stimulates bowel movements normalizing stool frequency in constipated patients (Quigley 2011). It should be noteworthy that most of the evidence regarding the beneficial effect of prebiotics in constipation is derived from animal studies. Human trials are only conducted on small number of constipated subjects (Table 1). However, one recent trial of prebiotics in sixty constipated women reported that there was no significant difference in satisfaction in relief of constipation between prebiotic group and placebo group (Linetzky Waitzberg et al. 2012). Further adequately powered studies need to be carried out to draw more definitive conclusions.

**Table 1:** Summary of randomized controlled trials of prebiotics for the management of chronic constipation

References	Subjects	Intervention	Comparator	Author’s conclusion
Linetzky Waitzberg et al. (2012)	Patients n = 60 (control n = 32, Intervention n = 28)	Inulin	Placebo	Decrease the amount of pathological bacteria of the *Clostridium* genera
Bouhnik et al. (2004)	Patients n = 65 (control n = 32, Intervention n = 33)	Lactulose	Polyethylene glycol	Beneficial effects, an increase in faecal *bifidobacteria* counts
Li et al. (2013)	Mice n = 40	Deshipu stachyose granules (DSG)	Placebo	Facilitating intestinal peristalsis and fecal excretion, increasing beneficial intestinal bacteria and inhibiting pathogenic bacteria
Li et al. (2011)	Rats n = 90	Prebiotics (a combination of GOS, XOS, OF and inulin)	Placebo	Beneficial effects on constipation

### Probiotics

Probiotics are live or attenuated microorganisms defined as being capable of conferring health benefits on their host when they are given in sufficient quantities and administered continuously, beyond any inherent nutritional value. Probiotics have demonstrated beneficial effects in patients with constipation, making them increasingly used as alterative treatment options (Table 2). A recent systematic review of probiotics for the treatment of chronic constipation demonstrated that probiotics did lead to a significant improvement in the mean number of stools per week (Dimidi et al. 2014). Several mechanisms have been proposed by which probiotics may benefit chronic constipation: (1) probiotics may modify the altered intestinal microbiota in patients with constipation; (2) probiotic metabolites may alter gut sensation and motility function (Waller et al. 2011); (3) some probiotics may regulate the intraluminal environment, such as increasing the end products of bacterial fermentation, reducing luminal pH (Waller et al. 2011), etc.

**Table 2:** Summary of randomized controlled trials of probiotics for the management of chronic constipation

References	Population	Intervention	Comparator	Author’s conclusion
Tabbers et al. (2011)	n = 159 (control n = 80, intervention n = 79)	*B. lactis* DN-173 010	Acidified milk without probiotics	Increased stool frequency, but not statistically significant compared with control group
Coccorullo et al. (2010)	n = 44 (control n = 22, intervention n = 22)	*L. reuteri* DSM 17938	Identical placebo	Increased bowel frequency
Favretto et al. (2013)	n = 30 (control n = 15, intervention n = 15)	*B. lactis* Bi-07	Fresh cheese without probiotics	Beneficial effects
Yang et al. (2008)	n = 126 (control n = 63, intervention n = 63)	*B. lactis* DN-173010	Acidified milk without probiotics	Beneficial effects on stool frequency, defecation condition and stool consistency
Ishizuka et al. (2012)	n = 17 (cross-over design)	*B. lactis* GCL2505	Milk-like drink	Beneficial effects
Waller et al. (2011)	n = 100 (control n = 34, Intervention: high dose n = 33 low dose n = 33)	*B. lactis* HN019	Capsules with rice maltodextrin	Decreased whole gut transit time in a dose-dependent manner
Mazlyn et al. (2013)	n = 90 (control n = 43, intervention n = 47)	*L. casei* Shirota	Fermented milk without probiotics	Improvement in constipation severity
Riezzo et al. (2012)	n = 20 (cross-over design)	*L. paracasei* IMPC 2.1	Artichokes without probiotics	Beneficial effects
Koebnick et al. (2003)	n = 70 (control n = 35, intervention n = 35)	*L. casei* shirota	Beverage without probiotics	Beneficial effects on self-reported severity of constipation and stool consistency

*Bifidobacteria* and *Lactobacilli* are the generally recognized beneficial species with various health-promoting functions such as the production of SCFAs, stimulation of intestinal peristalsis and increasing the humidity of the fecal bolus (Ojetti et al. 2014). A large randomized controlled trial demonstrated that the intake of an effective amount of *Lactobacillus plantarum* and *Bifidobacterium breve*, or *Bifidobacterium lactis* is able to significantly relieve the evacuation disorders and hard stools in patients with constipation (Del Piano et al. 2010). The supplementation with *Lactobacillus casei Shirota* could significantly increase the frequency of defecations and the softness of the stool in IBS-C patients (Koebnick et al. 2003). A positive effect of *Lactobacillus reuteri* on bowel movement frequency has been confirmed in both adult and children patients with constipation (Wu et al. 2013). Evidence showed *L. reuteri* could promote both the frequency and velocity of colonic myoelectric motility complex (Barbara et al. 2005). One in vitro study indicated that *Escherichia coli* Nissle 1917 supernatants could enhance colonic contractility by direct stimulation of smooth muscle cells (Bar et al. 2009).

Some probiotics, such as *Bifidobacterium lactis* DN-173010 and *Bifidobacterium longum* could modify the metabolic activities of the colonic microbiota and improve lactose digestion in Chinese lactose-intolerant subjects (He et al. 2008). In children, *Lactobacillus* casei rhamnosus Lcr35 showed a favorable effect on relieving the symptoms of constipation (Bu et al. 2007). The combination of probiotics and prebiotics, which is named synbiotics, may provide synergistic effects. Synbiotic administration may improve the survival of probiotics and restore intestinal microbial balance, which may have positive effects on the treatment of constipation (Khodadad and Sabbaghian 2010). A meta-analysis conducted by Alexander C. Ford reported that synbiotics appeared to be more effective than placebo in chronic constipation. And the number needed to treat (NNT) with synbiotics in chronic constipation was 5 (95 % CI 3–14) (Ford et al. 2014).

Probiotics have been widely used nowadays for the treatment of constipation, however, concerns about the safety of probiotics still warrant further discussion. Probiotic administration may modulate the composition of the intestinal microbiota and consequently induce the alteration of metabolic activities and colonic immunological activities (Dai et al. 2014). Besides, the probiotic administration may transfer the antimicrobial resistance genes to the normal intestinal flora and pathogenic species (Drago et al. 2013).

### Fecal microbiota transplantation

Fecal microbiota transplantation (FMT), also known as “fecal bacteriotherapy” or “fecal infusion”, refers to the process of transplanting the functional flora from the stools of healthy donor to the gastrointestinal tract of recipient individual. It has been proposed as a therapeutic approach for chronic constipation by reestablishment of the wide diversity of intestinal flora. For example, one patient reported by Andrews et al. developed constipation and received FMT therapy. Two to three days following therapy, the patient’s stool frequency increased to 1–2 per day without laxative-use (Borody et al. 2004). Borody et al. administered FMT to 4 patients with chronic constipation. All 4 patients experienced immediate resolution of symptoms of constipation to 1–2 stools per day with an accompanying resolution of most associated symptoms such as episodic nausea, vomiting, bloating and abdominal pain. And this improvement persisted for 6–28 months of follow-up (Borody et al. 1989). Li et al. treated 24 patients with slow transit constipation (STC) by means of FMT. The patients’ stool frequency increased from a mean of 1.8 (SD 1.3) per week to 4.1 (SD 2.6) at week 12 post-FMT. Compared with baseline, significant overall improvements were seen in gastrointestinal quality-of-life index score during 12 weeks of follow-up (Tian et al. 2016). Though published reports on FMT are few in number and consist of just small uncontrolled open studies and case reports, the therapeutic benefits of FMT for the treatment of constipation suggests potential for this inexpensive and safe treatment modality to undergo further investigations for clinical use.

## Conclusions

Collectively, the altered intestinal microbiota may play an essential role in the pathogenesis of chronic constipation. However, the precise mechanism of intestinal microbiota on the regulation of gut sensory and motor functions is still partly unclear. Future research identifying precisely how the intestinal microbiota participate in the modulation of gut physiology and pathophysiology will be beneficial for our understanding of the interactions between the intestinal microbiota and gut function, which is also invaluable in ultimately developing new therapeutic tools for the management of chronic constipation.

## Abbreviations

SCFAs: short-chain fatty acids
IBS-C: constipation predominant-irritable bowel syndrome
MMC: migrating myoelectric complex
5-HT: 5-hydroxytryptamine
SUS: starch utilization system
SIBO: small intestinal bacterial overgrowth
TGF-β: transforming growth factor-β
TNF-α: tumor necrosis factor α
FMT: fecal microbiota transplantation
STC: slow transit constipation
